# Close Canine Contact: A Case of Infantile Pasteurella Meningitis

**DOI:** 10.7759/cureus.40710

**Published:** 2023-06-20

**Authors:** Rachel T Cahn, Melissa Winkie, Collin John, Joseph D Lynch

**Affiliations:** 1 Dermatology, West Virginia University School of Medicine, Morgantown, USA; 2 Pediatrics, West Virginia University, Morgantown, USA; 3 Pediatrics and Internal Medicine, West Virginia University, Morgantown, USA

**Keywords:** pediatric hospital medicine, pediatric infectious disease, pasteurella infections, pasteurella infection, febrile infant, infantile fever, meningitis

## Abstract

This case report presents an interesting and rare cause of meningitis in young infants, *Pasteurella multocida*. Not only is the organism unusual but the well appearance of the infant made this diagnosis surprising. The 2021 American Academy of Pediatrics (AAP) clinical practice guidelines on well-appearing febrile infants brought a national guideline on the evaluation and management of fever in infants. However, providers should be aware that there is room for shared decision-making and that the guideline will miss a very small percentage of well-appearing infants with invasive bacterial infections.

## Introduction

*Pasteurella multocida* is a rare cause of meningitis in neonates and young infants [1-5]. Typical cases present with symptoms including poor feeding, irritability, emesis, and/or fever [1-5]. We present a unique case of an asymptomatic febrile infant who was admitted for workup of invasive bacterial infection. He was ultimately diagnosed with *Pasteurella multocida* meningitis. He was treated with intravenous ceftriaxone for three weeks and recovered fully without complications. This case highlights the subtlety in which serious bacterial infections can present in infants and the importance of timely identification. With the publication of the 2021 American Academy of Pediatrics (AAP) clinical practice guidelines for the management of well-appearing febrile infants, we also discuss the use of clinical guidelines to aid in decision-making and special considerations with rural populations.

## Case presentation

A 33-day-old male was admitted to our hospital for an infectious workup following a fever of 38.9°C (rectally obtained) found incidentally at a well-child check. He was born at 35 weeks via spontaneous vaginal delivery and had no complications post-birth. Approximately one week prior to admission, his mother had noted that he had been slightly fussier than usual. He was evaluated by his pediatrician at that time, and the fussiness was attributed to increased gassiness following feedings. He was prescribed a probiotic, and his fussiness improved. He then presented for his well-child check where the fever was noted, and the child was directly admitted to our hospital for further evaluation.

On admission, the patient had an unremarkable physical examination, with a non-bulging fontanelle, and consolable fussiness consistent with age. There were no sick contacts at home, and the child was not in daycare per the mother. A limited sepsis workup was undertaken given the infant’s age, fever, and well appearance with no evident source of infection. Laboratory results were remarkable for a white blood cell count (WBC) of 17.5 × 10^3^ cells/µL with elevated absolute neutrophil count (ANC) of 9,630 cells/ µL and an elevated C-reactive protein of 34 mg/L (normal range: <8 mg/L). Given the patient’s high inflammatory markers and elevated WBC, a more in-depth sepsis workup was initiated. A lumbar puncture (LP) was completed, and the cerebrospinal fluid (CSF) analysis was concerning for bacterial meningitis with 5,900 nucleated cells/µL, protein of 224 mg/dL, glucose of 1 mg/dL, and red blood cells (RBC) of 3/µL. The patient was started on intravenous (IV) vancomycin, ceftriaxone, and acyclovir for empiric treatment of meningitis. A Gram stain of the CSF did not show any organisms, but the cultures grew *P. multocida* within 22 hours of collection. Blood and urine cultures were negative throughout admission. The patient’s empiric antibiotic regimen was narrowed to IV ceftriaxone based on susceptibilities, 48 hours after culture collection. Further history gathered from the parents revealed that the family had two pet dogs who frequently licked the patient to give him “kisses.” Besides the dogs, the family did not have any other pets. The parents denied any dog bites or scratches on the patient.

On hospital day 3, the WBC count peaked at 26.4 × 10^3^ cells/µL with a neutrophilic predominance and subsequently trended down. A repeat LP was performed on day 4 of admission, and CSF analysis showed 255 nucleated cells/µL, protein of 238 mg/dL, glucose of 11 mg/dL, RBCs of 2/µL, and no growth by culture. The patient defervesced on hospital day 4 and remained afebrile for the remainder of admission. The patient was treated with a course of IV ceftriaxone for a total of 21 days and did well throughout his hospital stay with no complications. Upon follow-up two weeks after discharge, he continued to grow and develop appropriately and had a normal hearing screen. He has been doing well since discharge, meeting milestones, and having no adverse neurological sequelae.

## Discussion

*Pasteurella multocida* (*P. multocida*) is a Gram-negative coccobacillus that colonizes the oral mucosa of animals including those commonly kept as pets such as cats and dogs. Although it is most associated with cellulitis following a dog or cat bite, it is also a rare cause of meningitis and sepsis [3,4,6]. Only 38 cases of *Pasteurella* meningitis have been reported in infants since 2013 [2]. In contrast to cellulitis associated with *P. multocida*, meningitis and sepsis in neonates are usually the result of an atraumatic exposure to cats and dogs, such as licking or sniffing. Rarely there have been cases of horizontal and vertical transmission [4,7]. Symptoms of *P. multocida* meningitis are often nonspecific and can include poor feeding, emesis, irritability, and/or fevers. Complications include seizures, hemiparesis, brain abscess, hydrocephalus, and death [2]. *Pasteurella multocida* is typically susceptible to penicillin and later-generation cephalosporins, although it has resistance against cephalexin and clindamycin [1].

This case presentation of *P. multocida* meningitis highlights several important factors, the first of which is the critical role that primary care providers play in recognizing early signs of potentially life-threatening infections in infants. The only abnormal finding initially present was a fever of 38.9°C (rectally obtained), which was found incidentally during a routine visit. Without the swift admission facilitated by the patient’s pediatrician, treatment could have been significantly delayed.

The second factor that this case highlights is the importance of applying established criteria to guide initial workup while also taking into consideration individual patient situations. Per the 2021 AAP guideline, the patient’s elevated CRP suggests considering obtaining an LP, although this is a weak recommendation for infants 29-60 days in part due to the low prevalence of meningitis in published studies among this age group (0.25%) [6]. In contrast, following the Rochester criteria, the LP was performed and ultimately led to his diagnosis. The Rochester criteria were a guideline used prior to the 2021 AAP guidelines to identify infants at low risk of serious bacterial infection. If infants met all criteria, a limited sepsis workup could be undertaken, and if not, a more thorough assessment would be done. Because the 2021 guidelines have a less definitive recommendation for pursuing LP, this leaves the decision in the hands of the clinician and the guardians of the patient. The appearance of the infant in conjunction with laboratory abnormalities (or lack thereof) and risk toleration both by parents and medical providers should be considered when making diagnostic decisions that are not clearly defined by the guidelines. Shared decision-making is important in today’s medical landscape, and knowledge of current rates of infection is important to share with parents.

For those practicing in states with a large rural population such as West Virginia, it is important to consider the patient’s access to medical facilities when considering disposition and diagnostic evaluation. Limited access to pediatric care can and should influence clinical decisions made in the evaluation of febrile infants. For example, it would be less feasible to observe the patient from home if the family lived several hours away with limited access to medical care in the surrounding area.

## Conclusions

This case emphasizes the need for a high index of suspicion for potentially life-threatening infections in infants with febrile illnesses. The primary care provider played a vital role in her immediate recognition of an ominous symptom in this patient, allowing him to be promptly worked up and treated before his illness progressed. While the 2021 AAP clinical practice guidelines have streamlined the evaluation of well-appearing febrile infants between the ages of eight and 60 days, there remains a small subset of the population evaluated who will have invasive bacterial infections and will require deviation from the guidelines for definitive diagnosis. This highlights the importance of clinical decision-making and shared decision-making when using guidelines in practice, as none are perfect. Furthermore, this case shows that even something seemingly innocent like a dog “kiss” can result in serious illness in young infants.
